# Marine Natural Compounds with Biomedical Potential

**DOI:** 10.3390/biom12091242

**Published:** 2022-09-06

**Authors:** Elena Leychenko

**Affiliations:** G.B. Elyakov Pacific Institute of Bioorganic Chemistry, Far Eastern Branch, Russian Academy of Sciences, 159, Pr. 100 let Vladivostoku, Vladivostok 690022, Russia; leychenko@gmail.com

Marine organisms are an inexhaustible source of natural compounds that are promising for the creation of drugs or biologically active additives, which is closely related to the huge biological diversity of the resources of the World Ocean. More than 99% of the studied marine natural compounds are new ones, having no analogues among land plants and animals. The spectrum of biological activities demonstrated by metabolites of marine origin is extremely wide; they exhibit antitumor, cancer-preventive, analgesic, antimicrobial, neuroprotective and other actions. The probability of the release of new highly active substances from marine organisms, on the basis of which drugs for medicine and veterinary medicine can be created and the corresponding technologies for their use, is quite high.

Five contributions were published in this Special Issue, including two reviews and three original research articles which are devoted to study both low molecular weight compounds and bioactivity peptides having marine original.

In Ahmed M. Elissawy with co-authors’ review [1] collected, classified and described the study of marine sponge alkaloids, a rather complex and very diverse class of chemical compounds. To collect information, the authors used both various databases of experimental works and already published reviews. The authors’ main task was to identify and draw attention to alkaloids that exhibit strong cytotoxicity in vitro and, therefore, can be the basis for drug design. In total, about 240 molecules belonging to 20 different structural classes of alkaloids were described the properties and characteristics of representatives of each class were considered in detail. In this review, the evaluation of the cytotoxicity of alkaloid compounds extracted from marine sponges was carried out using the most common panels of cytotoxic cell lines, consisting of human breast cancer, colorectal carcinoma, alveolar and lung adenocarcinoma, lung fibroblasts, leukemia, and many others. This screening is a reliable tool for identifying molecularly targeted drugs and improving compounds that are candidates for anticancer drugs. In addition, the authors were able to identify patterns and rank marine sponge species according to the production of highly cytotoxic alkaloids. It has been shown that various genera belonging to the phylum porifera are a rich source of alkaloid molecules, including *Agelas* sp., *Suberea* sp., *Mycale* sp., *Haliclona* sp., *Epipolasis* sp., *Monanchora* sp., *Crambe* sp., *Reniera* sp., *Xestospongia* sp. and others.

The article by Maria Orfanoudaki et al. [2] studied the chemical composition of two Mediterranean demosponges, *Aplysina aerophoba* and *Spongia* sp., from which 20 secondary metabolites were isolated by various chromatographic methods. Five out of fifteen compounds isolated from *A*. *aerophoba* were novel natural products. The structures of the substances were confirmed by NMR and HRMS, they were mainly prenylated hydroquinones from *Spongia* sp. and halogenated compounds from *A. aerophoba*. Cytotoxic properties of all isolated compounds to T24 bladder and AGS stomach tumor cells (nine different cell lines were used) were determined using the common MTT assay. For AGS stomach tumor cells, 2-heptaprenyl-1-4-hydroquinone from *Spongia* sp. was found to be the most toxic with an IC_50_ value of 0.99 µM, while 2,6-dibromo-4-hydroxy-4-methoxycarbonylmethylcyclohexa-2,5-dien-1-one from *A. aerophoba* showed the highest cytotoxic activity in all other cell lines tested.

The article by Timofey V. Malyarenko et al. [3] was another experimental study of marine low molecule weight compounds with biomedical potential. The authors from Elyakov Pacific Institute of Bioorganic chemistry continued to study secondary metabolites and isolated six triterpene glycosides from the ethanol extract of the Far Eastern starfish *Solaster pacificus*, including three new pacificusosides A–C. Pacificusoside A contains a unique structure fragment of particularly an aldehyde group of side chains in its triterpene aglycon that has not previously been found in the sea cucumber triterpene glycosides or starfish steroidal glycosides. The other structures have closely related characteristics to sea cucumber glycosides. All compounds were tested against cancer cells (four cell lines were used) using MTS assay. Pacificusoside C and cucumariosides C_1_ and C_2_ suppressed the colony formation of the HT-29, RPMI-7951, and MDA-MB-231 cells at a nontoxic concentration of 0.5 µM.

Marine organisms are a unique source of not only low molecular weight bioactive compounds, but also peptide molecules with biomedical potential. In the review by Kasheverov et al. [4], using different ligands to acetylcholine receptors as the example, it was shown that various compounds of marine origin can be successfully used both as tools for studying biological targets and for drug design. α-Conotoxins were shown to be delicate tools for distinguishing between muscle and neuronal nAChRs, which is crucial for fundamental research on nAChRs, including the study of the mechanisms of opening and closing of channels. The authors presented detailed information on the results obtained in the last decade using such compounds in nAChR studies with computer modeling, synthetic analogues and mutants of receptors, X-ray and electron microscopy analyzes of complexes with nAChR and their models, which are acetylcholine-binding proteins and heterologously expressed ligand-binding domains.

Sea anemones are perhaps the leaders among marine animals in terms of the diversity of biologically active peptides. The article by Michela L. Mitchell et al. [5] was devoted to the study of the insulin-like peptide IlO1_i1, identified among transcripts similar to the insulin family, in the tentacle transcriptomes of the sea anemone *Oulactis* sp. Studies of the biological activity of IlO1_i1 synthesized by the solid-phase peptide synthesis were carried out on human insulin and insulin-like growth factor receptors. IlO1_i1 did not bind to insulin or insulin-like growth factor receptors, but exhibited weak activity against Kv1.2, 1.3, 3.1, and 11.1 (hERG) channels, as well as Nav1.4 channels. The role of insulin and insulin-like peptides (ILPs) in vertebrate animals is well studied, whereas the function of ILPs in sea anemones and their sister taxa yet to be seen. The authors demonstrated that the novel ILPs presented in sea anemone tissues perform specific biological functions (e.g., tentacles for defense), and that different ILPs can be localized to specific tissues. Further research needs to be conducted to establish, and to determine whether their activities correlate with specific biological functions in their tissue of origin.

Thus, this Special Issue contains a lot of new and useful information regarding marine natural compounds-potential drugs and indispensable tools for fundamental scientific research.
